# Complete Genome Sequencing of Protease-Producing Novel *Arthrobacter* sp. Strain IHBB 11108 Using PacBio Single-Molecule Real-Time Sequencing Technology

**DOI:** 10.1128/genomeA.00346-15

**Published:** 2015-04-23

**Authors:** Shashi Kiran, Mohit K. Swarnkar, Mohinder Pal, Rishu Thakur, Rupinder Tewari, Anil Kumar Singh, Arvind Gulati

**Affiliations:** a Plant Pathology and Microbiology Laboratory, Hill Area Tea Science Division, CSIR-Institute of Himalayan Bioresource Technology, Palampur, India; b Division of Biotechnology, CSIR-Institute of Himalayan Bioresource Technology, Palampur, India; c Department of Microbial Biotechnology, Panjab University, Chandigarh, India

## Abstract

A previously uncharacterized species of the genus *Arthrobacter*, strain IHBB 11108 (MCC 2780), is a Gram-positive, strictly aerobic, nonmotile, cold-adapted, and protease-producing alkaliphilic actinobacterium, isolated from shallow undersurface water from Chandra Tal Lake, Lahaul-Spiti, India. The complete genome of the strain is 3.6 Mb in size with an average 58.97% G+C content.

## GENOME ANNOUNCEMENT

The genus *Arthrobacter* belongs to high GC-content *Actinobacteria* established within the family *Micrococcaceae* (1, 2). Members of the genus *Arthrobacter* exhibit G+C content ranging from 59 to 66 mol% (3). More than 84 species have been described from alpine, Antarctic, forest, field, and rhizosphere soils; Antarctic lake and deep-sea sediments; South China Sea and Pohang basin waters; and extreme environments, including air in the Russian space laboratory, alpine glacier cryoconite, and an alpine ice cave (http://www.bacterio.net/arthrobacter.html). Members of this genus are involved in the biodegradation of the plant alkaloids nicotine (4), 2,4-dinitrotoluene (5), and 4-fluorocinnamic acid (6), and in the production of the enzymes isonitrile hydratase (7), serine hydroxymethyltransferase (8), beta-galactosidase (9), and dehydrogenases (10). During exploration of microbial communities from sediment and water samples collected from Chandra Tal Lake (32.29°N 77.36°E), an off-white, psychrotrophic isolate showing hydrolysis of skimmed milk was characterized as a novel species based on the highest identity of 97.46% from a 16S rRNA gene sequence comparison with *Arthrobacter russicus* GTC 863^(T)^.

The genomic DNA from a 72-h-old culture on tryptone soy agar (HiMedia, India) was extracted using the GenElute bacterial genomic DNA isolation kit (Sigma-Aldrich, USA). The genomic DNA was assessed for quality and quantity using NanoDrop 2000 (Thermo Scientific, USA) and Qubit version 2.0 fluorometer (Invitrogen, USA), respectively. Ten micrograms of genomic DNA was fragmented to 10-kb inserts using Covaris g-Tubes (Covaris Inc., USA). The genomic DNA library was prepared using a PacBio SMRTbell template preparation kit version 1.0. An SMRTbells template library was quantified and the quality of the sheared DNA was checked with a Bioanalyzer DNA 12000 chip (Agilent Technologies, USA). The sequencing of two SMRT cells with 180-min movie times was performed on a PacBio RS II system using P5 polymerase and C3 sequencing chemistry. The sequencing run generated 1,197,626,978 bases within 412,201 reads (*N*_50_ size 3,393 and mean subread length 2,905). These subreads were *de novo* assembled using the HGAP (hierarchical genome assembly process) protocol version 2.0 in SMRT Analysis version 2.2.0 (Pacific Biosciences, USA) and produced a complete circular genome sequence without gaps, with high coverage (282×) (11). The genome size was 3,595,718 bp with 58.97% G+C content and a 3.716-kb plasmid (55.32% G+C content) was also identified. The functional annotation performed on the Rapid Annotations using Subsystems Technology (RAST) server (12) predicted 3,454 genes for protein coding (CDSs), 46 genes for tRNAs, and 8 genes for rRNAs; 379 RAST subsystem categories were functionally assigned through the predicted genes. The comparison of the *Arthrobacter* sp. strain IHBB 11108 genome sequence performed using the RAST server with available genome sequences showed highest relatedness with *Renibacterium salmoninarum* ATCC 33209 (score 541) and *Arthrobacter chlorophenolicus* A6 (score 349). The annotation also predicted gene-coding clusters for protease enzyme, secretory serine protease (nine genes), cysteine protease (five genes), trypsin-like serine protease (two genes), putative protease (one gene), and Protease II (one gene).

### Nucleotide sequence accession numbers. 

The complete genome and plasmid sequences of *Arthrobacter* sp. strain IHBB 11108 have been deposited at DDBJ/EMBL/NCBIGenBank under the accession numbers CP011005 and CP011006. The strain has also been deposited under the accession number MCC 2780 at the Microbial Culture Collection (MCC) at the National Centre for Cell Science, Pune, India.
